# Body-Weight Gain in Women During Smoking Cessation Is a Sex-Specific Predictor of 6-Month Abstinence: A Retrospective Cohort Study

**DOI:** 10.3389/fpubh.2022.872220

**Published:** 2022-05-13

**Authors:** Chin-Wei Kuo, Chung-Fu Lin, Chuan-Yu Chen, Ru-Hsueh Wang, Chieh-Ying Chou, Hsiang-Ju Cheng, Jin-Shang Wu, Chang-Wen Chen, Chi-Chang Shieh, Tsung Yu

**Affiliations:** ^1^Division of Pulmonary Medicine, Department of Internal Medicine, National Cheng Kung University Hospital, College of Medicine, National Cheng Kung University, Tainan, Taiwan; ^2^Institute of Clinical Medicine, College of Medicine, National Cheng Kung University, Tainan, Taiwan; ^3^Department of Family Medicine, National Cheng Kung University Hospital, College of Medicine, National Cheng Kung University, Tainan, Taiwan; ^4^Health Promotion Association, National Cheng Kung University Hospital, College of Medicine, National Cheng Kung University, Tainan, Taiwan; ^5^Department of Public Health, College of Medicine, National Cheng Kung University, Tainan, Taiwan

**Keywords:** smoking, smoking cessation, failed abstinence, female, body weight

## Abstract

**Background:**

Smoking behavior differs between the sexes. Weight control is one of the main reasons leading to tobacco abuse in women but not in men. Studies on the predictive factors of cessation failure between sexes are scarce. This study is aim to investigate whether there are sex differences in the effect of weight gain on smoking cessation rate.

**Methods:**

Participants in the smoking-cessation program at a Medical Center in Taiwan between 2018 and 2019 were included. Details of age, sex, comorbidities, depression screening, nicotine dependence, body weight, and cessation medications of the participants were collected. The participants were classified based on their sex, and multivariable logistic regression analyses were conducted. Multivariable logistic regression analyses were performed for sensitivity analysis after stratifying the participants according to their weight loss (weight loss ≥ 1.5 kg and weight loss ≥ 3.0 kg).

**Results:**

A total of 1,475 participants were included. The body-weight gain in women was associated with failed abstinence (adjusted odds ratio (OR): 3.10, 95% CI: 1.10–9.04). In contrast, body-weight gain in men was associated with successful 6-month prolonged abstinence (adjusted OR: 0.77, 95% CI: 0.61–0.98). The adjusted ORs for any body-weight loss, body-weight loss ≥1.5 kg, and body-weight loss ≥3.0 kg were 0.28 (95% CI: 0.09–0.88), 0.14 (95% CI: 0.03–0.55), and 0.03 (95% CI: 0.01–0.42), respectively.

**Conclusion:**

Body-weight gain in women during a hospital-based smoking-cessation program is associated with abstinence failure. Further multicenter studies, including participants of different races and cultural backgrounds, are warranted.

## Introduction

Cigarette smoking is a widespread unhealthy behavior that leads to several diseases and premature death (1). Although the tobacco control policy reduced the worldwide prevalence of smoking, 9.5% of women were found to be active smokers in 2015 (2) indicating that women's smoking is an important public issue. Men and women smokers vary significantly in their physiological, cultural, and behavioral factors (3). For example, most men smoke for the pleasure and enjoyment of smoking, while women smoke to relieve stress and control their body weight (BW) (4, 5). Smoking activates dopamine in the ventral striatum of men, and the discrete subregion of the dorsal putamen of women (6). Plasma and saliva cortisol levels have predicted smoking relapse in men but not in women (7). Although the prevalence of women smokers is much lesser than that of men smokers (2), the former are more likely to get smoking attributable diseases, even under controlled tobacco exposure (8, 9) In addition, smoking during pregnancy is associated with poor birth outcomes (10) and early onset of smoking in children (11).

Abstinence is the most important way to reduce the harm caused by smoking. Studies have shown that women face greater difficulty in quitting smoking as compared with men (12). One plausible reason is that women are more likely to continue smoking for controlling their BW. Body-image challenges motivate young women to smoke (13), and smoking cessation is often associated with an increase in BW. A previous meta-analysis inferred that after 12 months of abstinence from smoking, 84% of individuals gained 4 to 5 kg mean BW (14). However, during the cessation program smoking abstinence-related negative mood and greater motivation to relieve distress from withdrawal symptoms were reported more in women than in men (15), suggesting that the barriers to smoking cessation differ between the sexes.

To the best of our knowledge, studies on the predictive factors of cessation failure between different sexes are scarce. In this study, we hypothesized that the predicted factor of failed smoking-cessation differs between the sexes and that the BW gained by women during the cessation program is associated with failed abstinence. Therefore, we performed a retrospective cohort study by using a well-registered, real-world in-hospital cessation program data from a Medical Center in the southern Taiwan to verify the hypothesis.

## Materials and Methods

### Study Design and Eligibility Criteria

We conducted a retrospective cohort study by collecting data from smokers who participated in smoking-cessation programs at the National Cheng Kung University Hospital (NCKUH) from 1 January 2018 to 30 November 2019. Participants aged ≥ 20 years and who received one or more cessation counseling in the program were included. The exclusion criteria were: (1) missing information on their demographics, smoking history, and follow-up smoking status, and (2) lost to follow-up when assessing the 6-month prolonged smoking abstinence. We checked every missing data and tried to fill them by crosslinking the electronic medical record and smoking cessation databank of NCKUH before excluding the participants. The first program was selected to analyze if the subjects participated in two or more cessation programs during the study period. Before commencing, the study was approved by the Institutional Review Board of NCKUH (ID number: B-ER-110-126).

### Variables and Outcome

We collected the participants' clinical information, including age, sex, marital status, underlying comorbidities (cardiovascular disease, cancer, cerebral vascular disease, diabetes mellitus, hypertension, liver disease, respiratory system disease, and renal disease), BW on each return to the clinic, site of cessation program initiation, smoking cessation history, Patient Health Questionnaire-2 (PHQ-2) depression screening test score (16, 17), Fagerstrom Test for Nicotine Dependence (FTND) score (18), type and duration of received cessation medication, and 6-month prolonged smoking abstinence of the participant from the electronic medical record and databank of tobacco-cessation of NCKUH. PHQ-2 is a 4-level, 2-item screening tool for major depressive disorder (MDD). The screening accuracy of the Chinese version of PHQ-2 had been validated. With a PHQ-2 score ≥ 2, the sensitivity and specificity of MDD are 0.88 and 0.82, respectively (17). The Fagerström Test for Nicotine Dependence (FTND) is a self-reported instrument that measures dependence through physiological and behavioral symptoms (19). FTND score ≥ 6 is defined as high-nicotine dependence (19). The outcome of this study was a 6-month prolonged abstinence rate, which is referred to as the rate of participants' sustained abstinence after the initial grace period until the 6-month follow-up (20). The grace period is 12 weeks in this study.

### Tobacco-Cessation Program in NCKUH

In 2002, the Taiwan Bureau of Health Promotion launched a nationwide smoking-cessation program. The participating institutions received grant support to implement the program and record normative individual-level registration data (21). Active smokers who visited our hospital would be referred to the tobacco-cessation team, which consisted of government-certified health education nurses, a chest physician, and a family physician. A 12-week tobacco-cessation program would be initiated under the agreement of participants. The cessation program could be initiated from in-hospital or out-patient services. The hospital-initiated cessation program followed the Ottawa model (22) and the clinical smoking cessation services guidelines of Taiwan (23), which were adapted from the published guidelines from the Department of Health and Human Services of the United States with updates (24) providing health education, behavioral intervention, and cessation medications. The out-patient cessation services were performed by health education nurses and medical doctors, giving the individual cessation counseling and medication. On the first visit of each participant, health education nurses recorded the participants' smoking history, PHQ-2 score, and FTND score (18). All the participants were recommended to receive pharmacotherapy for smoking cessation. Pharmacotherapy included nicotine replacement therapy (NRT; nicotine patch, nicotine gum, and nicotine inhaler) and non-NRT (varenicline and bupropion). The choice of medication was based on the preferences and contraindications of the participant. The dose of medication determined the participants' daily cigarette consumption (25–27). Participants were asked to return to the smoking cessation clinic every 1 or 2 weeks for the first 4 weeks, and every 3 to 4 weeks in the following 8 weeks. Upon the return, the smoking status, BW, and compliance with cessation medications would be recorded. The BW of the participants was measured using the same electric weighing scale on every visit. The status of the 6-month prolonged smoking abstinence was assessed *via* telephonic interviews. Participants who refused for telephone interviews or cannot be contacted were recorded as lost to follow-ups.

### Statistical Analysis

The characteristics of both the sexes were compared using a chi-square test or Fisher's exact test and are presented as numbers and percentages. The characteristics affecting the failure of smoking abstinence during the 6-months were analyzed by performing univariable and multivariable logistic regression analyses. Firth logistic regression was conducted for the analysis of binary outcomes with small samples. Characteristics include age (≥ 65 vs. < 65 years), sex (men vs. women), number of comorbidities (≥ 2 vs. < 2), PHQ-2 (≥ 2 vs. < 2), FTND score (≥ 6 vs. < 6), ever quit smoking for 6 months (yes vs. no), BW gain (yes vs. no), and cessation medications (only varenicline, only bupropion, only NRT, both NRT, and non-NRT vs. no use or use < 4 weeks) were considered covariables for the regression model. Because the cessation medications yield better outcomes when used for 4 weeks or longer (28), participants using these medications for < 4 weeks and those not using them were placed under the same category. For participants who received cessation medication for ≥ 4 weeks, they would be categorized into “only varenicline”, “only bupropion”, and “only NRT”. Whether participants received NRT and non-NRT concurrently or separately, they were classified into “both NRT and non-NRT groups. The association between covariable and failure of smoking abstinence for 6 months was presented using odds ratios (ORs) and 95% CIs. Two-sided *P*-values of < 0.05 were considered statistically significant. All the statistical analyses were conducted using the SAS software (version 9.4, SAS Institute, Cary, NC, USA).

### Sensitivity Analysis

To test the robustness of the effect of BW changes on smoking abstinence in different sexes, a multivariable logistic regression analysis was performed by changing the covariable from BW gain to BW loss. We used 1.5 and 3.0 kg as cutoff values based on the median and upper quartile of participant BW loss. We did not analyze the dose-response effect of BW gain because it might have been affected by the participants who withdrew from the cessation program on gaining slight BW.

## Results

**Figure 1:**
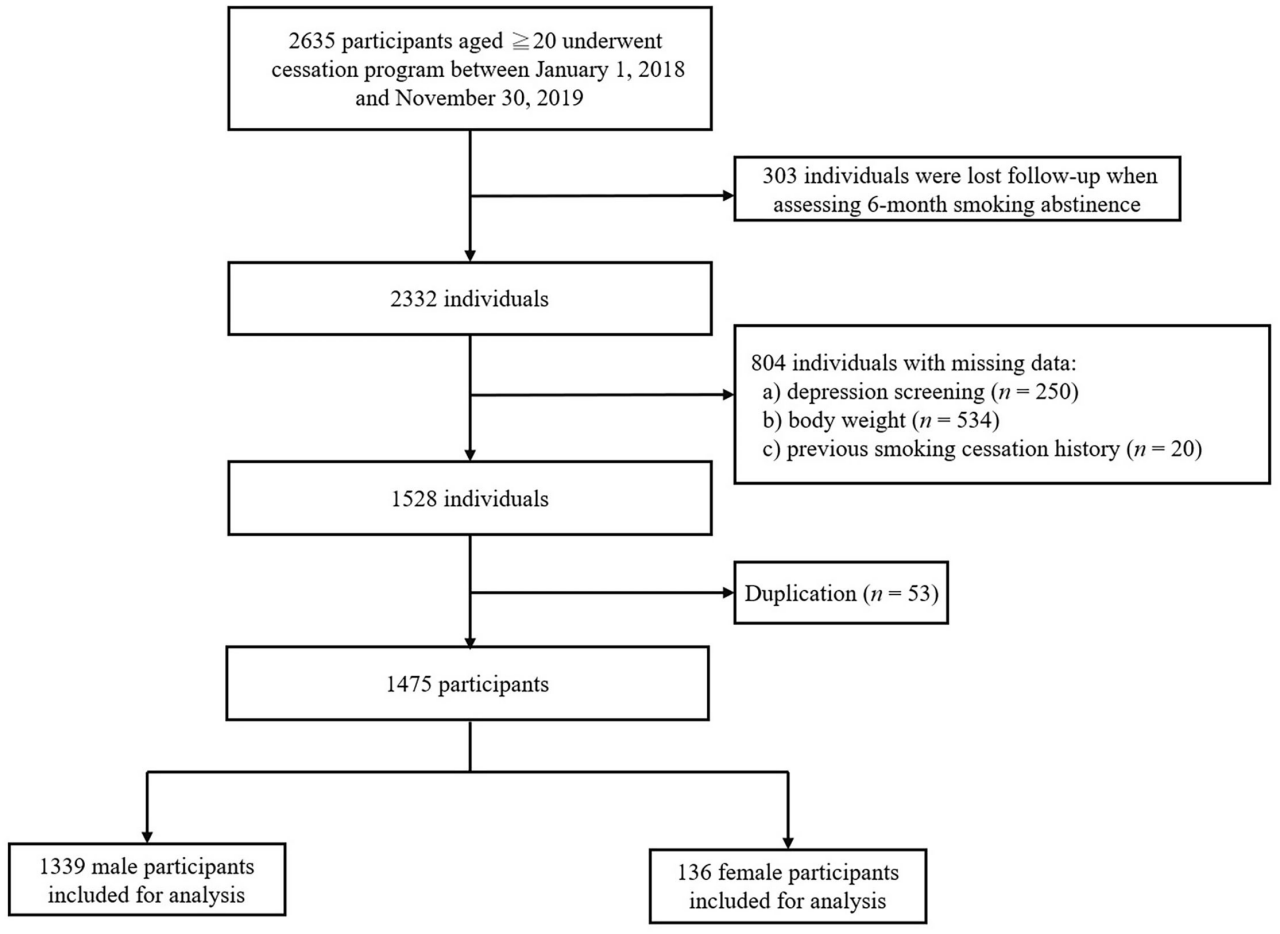
Flow chart for inclusion of participants.

Among the 2,635 individuals who participated in the smoking-cessation program during the study period, 303 participants were excluded as they were lost to follow-up when assessing the 6-month prolonged smoking abstinence, 804 participants were excluded because of their missing information, and 53 participants were excluded because of duplicated data. The remaining 1,475 participants were included in the analysis (Figure 1). Table 1 shows the demographic and clinical characteristics of 1,339 (90.8%) men and 136 (9.2%) women among a total of 1,475 participants. Compared with men, a larger proportion of the women were younger than 65 years, unmarried, had fewer comorbidities, PHQ-2 ≥ 2, and were initiated through outpatient service. The FTND score, BW gain during the program, and usage of types of cessation medication were similar for both the sexes. The 6-month prolonged abstinence rate of women was lower than that of men (21.3 vs. 36.7%, *P* < 0.001).

**Table 1:** Characteristics of smokers participating in a smoking cessation program.

	**Men (*n* = 1339)**	**Women (*n* = 136)**	***p*-value^b^**
Age (years)			0.008
<65	1063 (79.4)	121 (89.0)	
≥ 65	276 (20.6)	15 (11.0)	
Married			<0.001
Yes	966 (72.1)	74 (54.4)	
No	373 (27.9)	62 (45.6)	
Comorbidities^a^			<0.001
<2	841 (62.8)	112 (82.4)	
≥ 2	498 (37.2)	24 (17.7)	
PHQ-2 score			0.022
<2	1273 (95.1)	123 (90.4)	
≥ 2	66 (4.9)	13 (9.6)	
FTND			0.101
<6	429 (32.0)	53 (39.0)	
≥ 6	910 (68.0)	83 (61.0)	
Ever cessation for 6 months			0.009
Yes	209 (15.6)	33 (24.3)	
No	1130 (84.4)	103 (75.7)	
Site of initiation			<0.001
In-patient	886 (66.2)	64 (47.1)	
Out-patient	453 (33.8)	72 (52.9)	
BW gain during program			0.420
Yes	689 (51.5)	65 (47.8)	
No	650 (48.5)	71 (52.2)	
Cessation medications			0.637^c^
No use or use < 4 weeks	1100 (82.2)	112 (82.4)	
Only varenicline	80 (6.0)	7 (5.1)	
Only bupropion	7 (0.5)	2 (1.5)	
Only NRT	89 (6.6)	8 (5.9)	
Both NRT and non-NRT	63 (4.7)	7 (5.1)	
6 months of abstinence after program			<0.001
Yes	492 (36.7)	29 (21.3)	
No	847 (63.3)	107 (78.7)	

*BW, body weight; FTND, Fagerstrom test for nicotine dependence; NRT, nicotine replacement therapy; PHQ-2, Patient Health Questionnaire-2*.

a *Comorbidities include cardiovascular disease, cancer, cerebral vascular disease, diabetes mellitus, hypertension, liver disease, respiratory system disease, and renal disease*.

b *P-values were calculated by chi-square test except for cessation medication*.

c *Fisher's exact test*.

### Predictors of the Failed 6-Month Smoking Abstinence for All Included Participants

The results of the univariate and multivariable logistic regression analyses for all the included participants are shown in Table 2. The univariable analysis inferred a positive association with a failed 6-month prolonged smoking abstinence in participants who scored FTND ≥ 6 or used both NRT and non-NRT. On the contrary, covariates including those aged ≥ 65 years, men, married, comorbidities ≥ 2, ever cessation for 6 months, BW gain, in-patient initiated were negatively associated with failed abstinence. The multivariable analysis associated FTND ≥ 6 (OR: 1.99, 95% CI: 1.56 to 2.53) with a failed 6-month prolonged smoking abstinence, and negatively associated age ≥ 65 (OR: 0.59, 95% CI: 0.44 to 0.79), male (OR: 0.55, 95% CI: 0.35 to 0.86), married (OR: 0.59, 95% CI: 0.45 to 0.77), ever cessation for 6 months (OR: 0.54, 95% CI: 0.40 to 0.74), in-patient initiated (OR: 0.32, 95% CI: 0.23 to 0.43), with only varenicline usage (adjusted OR: 0.54, 95% CI: 0.32 to 0.92), and only NRT usage (adjusted OR: 0.59, 95% CI: 0.36 to 0.95) with a failed 6-month prolonged smoking abstinence. BW gain and PHQ-2 ≥ 2 unaffected 6-months prolonged smoking abstinence (*P* > 0.05).

**Table 2:** Univariable and multivariable logistic regression for predictors of failed 6-month smoking abstinence for all the participants (*n* = 1475).

	**Crude OR (95% CI)**	***p*-value**	**Adjust OR (95% CI)^a^**	***p*-value**
Age ≥ 65 (years)	0.46 (0.36–0.60)	<0.001	0.59 (0.44–0.79)	<0.001
Male	0.47 (0.31–0.71)	<0.001	0.55 (0.35–0.86)	0.010
Married	0.49 (0.38–0.62)	<0.001	0.59 (0.45–0.77)	<0.001
Comorbidities ≥ 2	0.65 (0.52–0.81)	<0.001	1.01 (0.79–1.30)	0.936
PHQ-2 ≥ 2	1.19 (0.73–1.94)	0.483	1.00 (0.59–1.69)	0.989
FTND ≥ 6	1.98 (1.58–2.48)	<0.001	1.99 (1.56–2.53)	<0.001
Ever cessation for 6 months	0.60 (0.45–0.79)	<0.001	0.54 (0.40–0.74)	<0.001
BW gain	0.80 (0.65–0.99)	0.042	0.83 (0.66–1.05)	0.113
In-patient initiated	0.38 (0.30–0.48)	<0.001	0.32 (0.23–0.43)	<0.001
Cessation medications				
No use or use <4 weeks	Reference		Reference	
Only varenicline	1.27 (0.79–2.03)	0.324	0.54 (0.32–0.92)	0.031
Only bupropion	0.71 (0.19–2.67)	0.615	0.40 (0.10–1.65)	0.205
Only NRT	1.01 (0.66–1.55)	0.965	0.59 (0.36–0.95)	0.012
Both NRT and non-NRT	2.09 (1.17–3.74)	0.013	0.93 (0.49–1.75)	0.814

*BW, body weight; CI, confidence interval; FTND, Fagerstrom test for nicotine dependence; NRT, nicotine replacement therapy; OR, odds ratio; PHQ-2, Patient Health Questionnaire-2*.

a *Adjusted for age, sex, marriage status, comorbidities, PHQ-2, FTND, cessation history, BW gain, site of program initiated, and cessation medication*.

### Predictors of the Failed 6-Month Smoking Abstinence Following Sex Stratification

**Figure 2:**
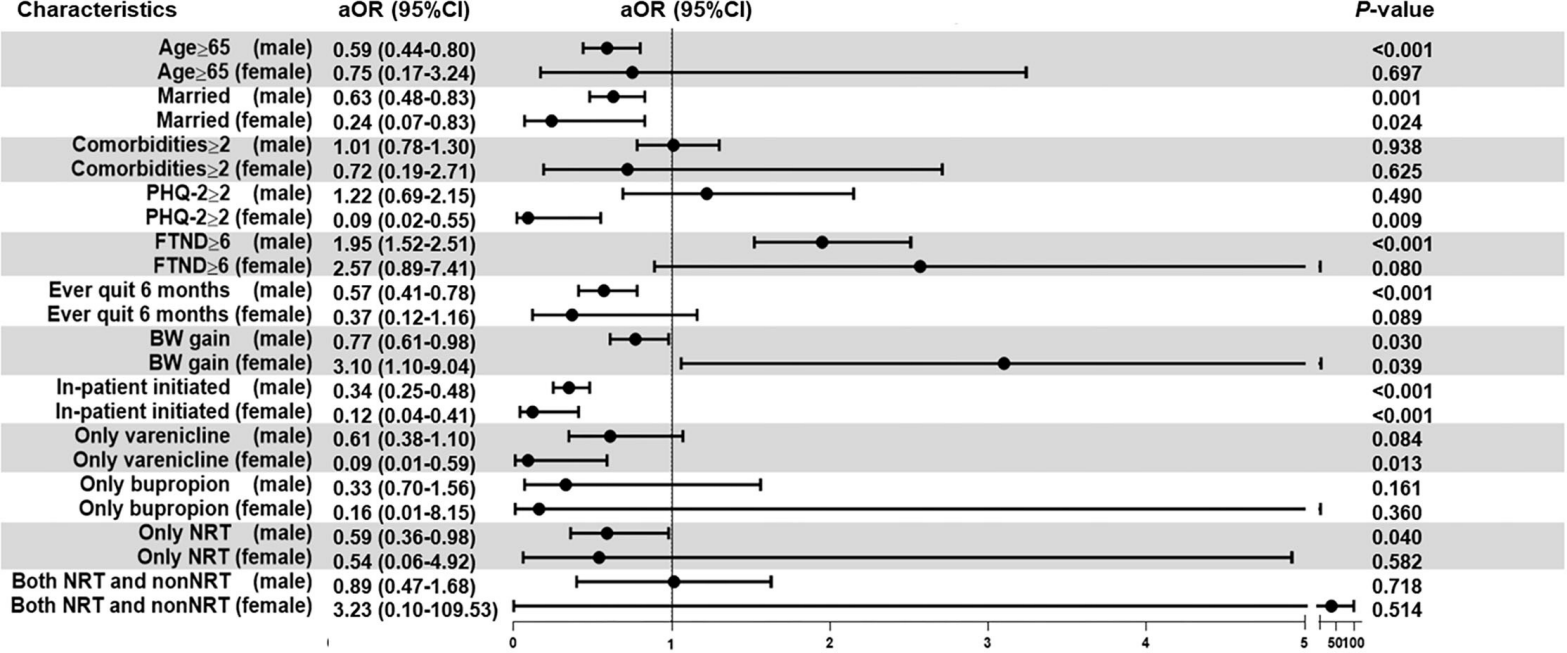
Multivariable logistic regression following sex stratification. The analyses were adjusted for age, sex, comorbidities, PHQ-2, FTND, cessation history, BW gain, site of program initiation, and cessation medication. Firth regression analyses were used for the women subgroup. Bold values indicate a statistically significant difference with a *p*-value of < 0.05. aOR, adjusted odds ratio; BW, body weight; CI, confidence interval; FTND, Fagerstrom test for nicotine dependence; NRT, nicotine replacement therapy; PHQ-2, Patient Health Questionnaire-2.

Figure 2 demonstrates the results of the multivariable analysis after gender stratification of the participants. The BW gain was positively associated with a failed 6-month prolonged smoking abstinence in the women (adjusted OR: 3.10, 95% CI: 1.06 to 9.04) and negatively associated in the men (adjusted OR: 0.77, 95% CI: 0.61 to 0.98). The PHQ-2 ≥ 2 (adjusted OR: 0.09, 95% CI: 0.02 to 0.55) and only varenicline use (adjusted OR: 0.09, 95% CI: 0.01 to 0.59) were negatively associated with a failed 6-month prolonged smoking abstinence in the women, and unassociated with smoking abstinence in the men. FTND ≥ 6 was associated with a failed smoking abstinence in men (adjusted OR: 1.95, 95% CI: 1.52 to 2.51). For men, age ≥ 65 years (adjusted OR:0.59, 95% CI:0.44 to 0.80), with only NRT usage (adjusted OR: 0.59, 95% CI: 0.36 to 0.95), and ever quit smoking for 6 months (adjusted OR: 0.57, 95% CI: 0.41 to 0.78) were associated with successful abstinence. In both the sexes, participants associated with a successful 6-month prolonged smoking abstinence were married and had participated through in-patient initiated programs.

### Sensitivity Analysis

The results of the sensitivity analysis are listed in Table 3. In the multivariable analysis, the BW loss during the cessation program was associated with a successful 6-month prolonged smoking abstinence for women by replacing the covariable of BW gain by BW loss (adjusted OR: 0.28, 95% CI: 0.09 to 0.88), BW loss ≥ 1.5 kg (adjusted OR: 0.14, 95% CI: 0.03 to 0.55), and BW loss ≥ 3.0 kg (adjusted OR: 0.03, 95% CI: 0.01 to 0.42). However, no statistically significant association was observed between BW loss and successful abstinence in men.

**Table 3:** Multivariable logistic regression for predictors of failed 6-month smoking abstinence after replacing covariable of body weight gain by body weight loss^a^.

	**Men (*****n*** **=** **1339)**		**Women (*****n*** **=** **136)^b^**	
	**Adjust OR (95% CI)**	***p*-value**	**Adjust OR (95% CI)**	***p*-value**
Any body-weight loss	0.94 (0.72–1.24)	0.669	0.28 (0.09–0.88)	0.029
Body-weight loss ≥1.5kg	0.78 (0.56–1.09)	0.141	0.14 (0.03–0.55)	0.005
Body-weight loss ≥3kg	0.69 (0.43–1.10)	0.115	0.03 (0.01–0.42)	0.010

*CI, confidence interval; OR, odds ratio*.

*^a^Multivariable logistic regression analyses that adjusted for age, sex, marriage, comorbidities, depression screen, daily cigarette smoke, the Fagerstrom test for nicotine dependence, motivation, ever cessation for 6 months, cessation medication, and in-hospital counseling*.

b *Firth regression was used for the women subgroup*.

## Discussion

Previous studies have found that smoking behavior differed between the sexes (3), and women smokers have more difficulty in quitting smoking than men smokers (13). To lower the prevalence of smoking among women, it is important to understand the factors associated with the failure of cessation in women smokers. However, studies investigating the predictors of failed smoking abstinence in hospital-based cessation programs are paucity. In this retrospective cohort study, data from 1,475 individuals were analyzed who had participated in a hospital-based smoking-cessation program. The collected data were considered credible because the factors, including age, sex, comorbidities, FTND scores, and cessation medications were monitored by the Taiwan Bureau of Health Promotion. In the multivariable logistic regression model adjusted for age, FTND score, PHQ-2, a cessation medication, other factors, and the BW gain was found to be associated with a failed 6-month prolonged smoking abstinence in women (OR: 3.10, 95% CI: 1.10 to 9.04) and with a successful 6-month prolonged smoking abstinence in men (OR: 0.77, 95% CI: 0.61 to 0.98). The sensitivity analyses showed that BW loss during the cessation program was associated with a successful 6-month prolonged smoking abstinence in women, but not in men. Thus, it was concluded that BW gain might be a sex-specific predictor of failed smoking abstinence in smoking-cessation programs.

Body-image dissatisfaction differs between the sexes. Women choose a smaller size whereas men choose a similar or larger size than their ideal body sizes (29, 30). In comparison with men, women have a higher risk of dissatisfaction with their body weight, report more disordered eating, and are less likely to recover their ideal BW from eating disorders (31, 32). Weight control is one of the main reasons leading to tobacco abuse in women (4, 5). On the other hand, weight gain might be one of the barriers in quitting smoking for women (33, 34). Cropsey et al. reported that women prisoners, who arguably have a stable energy intake and output, had average 10 pounds weight gain on the 6th month of smoking cessation, although the gain of body weight was decreased on the 12th month of cessation (35). The effect of smoking cessation on body mass index was higher for older women than for younger (36). In a previous smoking cessation study for postmenopausal women, the average weight gained was 6.5 kg (14 pounds) after 16 months of quitting smoking (37). To avoid failure of smoking abstinence, multidisciplinary weight control strategies (including education, calorie restriction, increasing physical activity, and cessation medication) should be introduced to the participants (38), especially women. Metanalysis concluded that pharmacological interventions limited the short-term weight gain during smoking cessation. However, there are no clinically effective interventions to restrict long-term weight gain currently (39).

Our study results showed that women participants with a PHQ-2 score ≥ 2 were associated with successful abstinence from smoking. However, previous research showed that depressive symptoms predicted a lower smoking abstinence rate, and there was no evidence of sex-specific effects of depression on smoking cessation (40, 41). PHQ-2 is a depression screening questionnaire rather than a diagnostic tool (17). Further investigation regarding PHQ-2 and smoking cessation in women is warranted.

Several limitations required to be acknowledged in this study. First, this study was conducted in Taiwan and body dissatisfaction differs based on cultural background, race, and economic status, (42) thus, the result of this study should be generalized with caution. Second, as this study was conducted retrospectively and the smoking history was dependent on the report of the participants, recall bias could not be excluded. However, other data including comorbidities, FTND, and cessation medication were regulated by the Taiwan Bureau of Health Promotion, so the robustness and accuracy of these data are supposed to be reliable. Third, apart from the information regarding the marital status of participants, information regarding active pregnancy, or child-bearing was unavailable. Although several pregnant women try to quit smoking due to the health concerns surrounding them and the fetus (3). A previous study had reported that among the few Taiwanese women who quit smoking during pregnancy, most relapsed in the 1st year after childbirth (43). Thus, the absence of pregnancy and child-bearing data should not affect the results of this study. Fourth, the women comprise only 9.2% of the participants. Given the large size of our study cohort, the sample size of women participants were sufficient. At last, the doses of NRT are different among the participants. However, the dose of NRT was determined by the participants' daily cigarette consumption in our study. This dosing strategy is widely used in the clinical trials and real-world conditions, and this limitation should not affect the results of our study.

## Conclusions

BW gain during a hospital-based smoking-cessation program is a sex-specific predictor of failed abstinence. Furthermore, multicenter studies including participants of different races and cultural backgrounds are warranted.

## Data Availability Statement

The original contributions presented in the study are included in the article/supplementary material, further inquiries can be directed to the corresponding author.

## Ethics Statement

The studies involving human participants were reviewed and approved by Institutional Review Board of NCKUH (ID number: B-ER-110-126). The Ethics Committee waived the requirement of written informed consent for participation.

## Author Contributions

C-WK, C-FL, and TY designed the study. C-WK, C-FL, C-YChe, C-WC, and C-CS searched the literature. C-WK, C-YChe, R-HW, C-YCho, H-JC, and J-SW collected the data. C-WK, C-FL, and TY did the statistical analysis. C-WK, C-FL, and C-YChe wrote the manuscript. All the authors contributed to and approved the final manuscript.

## Funding

This study was funded by grants from the National Cheng Kung University Hospital (NCKUH-11106011).

## Conflict of Interest

The authors declare that the research was conducted in the absence of any commercial or financial relationships that could be construed as a potential conflict of interest.

## Publisher's Note

All claims expressed in this article are solely those of the authors and do not necessarily represent those of their affiliated organizations, or those of the publisher, the editors and the reviewers. Any product that may be evaluated in this article, or claim that may be made by its manufacturer, is not guaranteed or endorsed by the publisher.
